# Our 1901 Meeting

**Published:** 1901-10

**Authors:** 


### OUR 1901 MEETING.

ATLANTIC CITY proved a veritable Mecca for the profession of America in September. More than three hundred journeyed to that attractive seaside resort, and there participated in one of the most important meetings ever held by the profession. Almost every section of our country save the Pacific coast was represented. The extreme North had a goodly number of delegates, while the Sunny South had the largest representation it has ever sent to a national convention. The Middle West had representatives from more than a dozen States, while the New England territory vied with the Middle States in the number of members and delegates present.

The programme had something to interest everyone who attended, and the position of the Association on the many serious problems confronting it was well defined in a series of resolutions which will be found in this number of the JOURNAL.

The programme again proved to be overloaded, but this was in part due to a lengthy consideration of several valuable Committee and State Secretary reports.

The clinics were well attended, and much interest was shown in them; but, as always before, the greatest interest was shown by local veterinarians or those within a fifty-mile radius of the convention city, and, much to the disappointment of all, many of these did not attend a single session of the convention, dropping in on the day of the clinics, thus further asserting the fact that these belong in the domain of State and local organizations.

